# Aur. Cor. Celsus on Medicine

**Published:** 1837-01

**Authors:** 


Art. VII.—*Aur. Cor. Celsus on Medicine, in eight Books, Latin and English. Translated from* L. Targa's *Edition, the words of the Text being arranged in the order of Construction. With explanatory notes, &c., designed to facilitate the Progress of Medical Students.* By Alexander Lee, a.m., Surgeon.—Two vols. 8vo. pp. 318, 350. *London,* 1831, 1836.

The title of this book explains its nature and object; and, after examining it, we are willing to admit that it will be very useful to the medical student who is imperfectly skilled in the Latin language, and wishes to improve his acquaintance with it. The "ordo" in the margin, after the manner of the Delphin editions of the Latin classics, cannot fail to afford the most important help to the tender grammarian, while the English translation at the bottom will render his progress at once easy, rapid, and safe. This, however, is the extent of the praise we can bestow on the work; as the translation, although generally conveying the meaning of the original author, cannot be said to be in any respect good. It is not close enough to have the merit and advantage of a strictly literal version; while it is still further removed from every claim to elegance, or even neatness. The author is apparently unqualified to produce anything like a classical English translation of his author; he ought therefore to have adhered to the literal interpretation of him : and it would have been easy for a good English scholar to have given a translation much more literal, and yet very superior in point of style. In this respect, Dr. Collier's translation has greatly the advantage of Mr. Lee's, although the work of the latter will be found much more serviceable to the class of students for whom it is intended.

